# Gadolinium-based contrast agent toxicity: a review of known and proposed mechanisms

**DOI:** 10.1007/s10534-016-9931-7

**Published:** 2016-04-06

**Authors:** Moshe Rogosnitzky, Stacy Branch

**Affiliations:** MedInsight Research Institute, Baltimore, MD 21202 USA; Center for Drug Repurposing, Ariel University, 40700 Ariel, Israel

**Keywords:** Gadolinium-based contrast agent (GBCA), Gadolinium toxicity, Gadolinium, Magnetic resonance imaging, Gadolinium toxicity mechanisms, Gadolinium chelation

## Abstract

Gadolinium chelates are widely used as contrast media for magnetic resonance imaging. The approved gadolinium-based contrast agents (GBCAs) have historically been considered safe and well tolerated when used at recommended dosing levels. However, for nearly a decade, an association between GBCA administration and the development of nephrogenic systemic fibrosis (NSF) has been recognized in patients with severe renal impairment. This has led to modifications in clinical practices aimed at reducing the potential and incidence of NSF development. Newer reports have emerged regarding the accumulation of gadolinium in various tissues of patients who do not have renal impairment, including bone, brain, and kidneys. Despite the observations of gadolinium accumulation in tissues regardless of renal function, very limited clinical data regarding the potential for and mechanisms of toxicity is available. This significant gap in knowledge warrants retrospective cohort study efforts, as well as prospective studies that involve gadolinium ion (Gd^3+^) testing in patients exposed to GBCA. This review examines the potential biochemical and molecular basis of gadolinium toxicity, possible clinical significance of gadolinium tissue retention and accumulation, and methods that can limit gadolinium body burden.

## Introduction

It was previously widely believed that GBCAs are rapidly and completely excreted from the human body in an intact state. In recent years, however, there is a rapidly growing body of data that demonstrate that gadolinium accumulates in tissues (including brain, bone, and kidneys) of patients exposed to GBCAs during magnetic resonance imaging (MRI), despite normal renal function. Retention of gadolinium increases in those who have repeated GBCA exposure. Patient-initiated survey results available through the Lighthouse Project (patient advocacy group) show the onset of a series of symptoms (including neurological, musculoskeletal, and dermal) in patients within a month of their last MRI. Given the increasing data available regarding tissue deposition in patients without renal impairment, the U.S. Food and Drug Administration (FDA) published a safety announcement in July 2015 that it is investigating the risk of brain deposits associated with the repeated use of GBCAs MRI (FDA Drug Safety Communication 2015).

Due to a lack of retrospective or prospective clinical cohort studies regarding the association of post-exposure medical conditions and recurrent GBCA exposure, the clinical significance of gadolinium tissue accumulation in patients without renal impairment is not fully known. Nonetheless, the potential for toxicity can be gleaned from published data of in vivo and in vitro studies of gadolinium toxicity, GBCA biochemistry, and differential gadolinium chelate stability. The understanding of the mechanisms of gadolinium toxicity can help determine the clinical significance of gadolinium retention in tissues so that the risks of GBCA use for MRI can be better assessed. This then can serve to develop guidelines for GBCA use, the possible development of new gadolinium chelates with higher stability and less toxicity, and determining the utility of gadolinium chelation therapy.

In 2006, Grobner (2006) established an association between NSF development and exposure to GBCAs during MRI. A number of published studies suggest several explanations for GBCA-related NSF development including, impaired clearance of gadolinium by the kidneys leading to tissue accumulation of dissociated Gd. More of the administered dose of gadolinium in those with severe renal dysfunction is available due to reduced elimination from the kidneys. The increased level of Gd^3+^ available leads to adverse effects including NSF. Those with normal renal function who undergo repeated GBCA administration will, over time, have higher levels of Gd^3+^ tissue deposition and potentially increase the risk of gadolinium-related toxicity. Studies done in vitro uncover a number of biochemical and molecular alterations that may be associated with adverse effects seen in vivo. Although some acute effects of GBCA exposure have been reported, including anaphylactic and other acute reactions (Blasco-Perrin et al. 2013; Terzi and Sokmen 1999; Unal and Arslan 1999), the potential for chronic and delayed manifestation of toxicity should be considered.

Toxicity of GBCAs has primarily been attributed to the dissociation of Gd^3+^ from the chelated complexes. This dissociation is believed to be related to differences in the stability of the complexes among the various types of GBCAs (Port et al. 2008; Sieber et al. 2008a). However, there is data that suggest that competitive chelation with components in the extracellular matrix may also play a role in the tissue distribution and toxicity of GBCAs (Taupitz et al. 2013). Non-complexed Gd^3+^ and other members of the lanthanide series (e.g., samarium, europium, and cerium) have long been known to deposit in bone tissue of animals and humans. However, a number of studies measuring Gd^3+^ deposition show that it can be found in other tissues including the skin, kidneys (Koeberl and Bayer 1992; Sieber et al. 2008b; Wang et al. 2015), liver (Sieber et al. 2008b; Wang et al. 2015), and brain (Darrah et al. 2009; Gibby et al. 2004; Koeberl and Bayer 1992; Murata et al. 2016). The non-chelated Gd^3+^ retained in body tissues can serve as a source of toxicity.

## Overview of gadolinium-based contrast agents

The chemical bonds in GBCAs are made of a gadolinium ion (the MRI active aspect) and a carrier molecule. A carrier molecule is called a chelating agent, which modifies the distribution of gadolinium within the body to overcome its toxicity while maintaining its contrast properties. Gadolinium-based contrast agents are used intravenously to enhance the detail and clarity of diagnostic MRI or magnetic resonance angiography. The current FDA and EMA-approved GBCAs are gadobenate (MultiHance), gadobutrol (Gadavist), gadodiamide (Omniscan), gadopentetate (Magnevist), gadoterate (Dotarem), gadoteridol (ProHance), gadoversetamide (OptiMARK), gadoxetate (Eovist), and gadofosveset (Ablavar, US only). These agents consist of a central paramagnetic Gd^3+^ chelated to a ligand to prevent direct toxicity by free Gd^3+^. Free Gd^3+^ is a toxic lanthanide heavy metal with a size similar to that of Ca^2+^. This similarity can lead to competitive inhibition of biological processes requiring Ca^2+^ and cause toxicity. Lanthanide ions such as Gd^3+^ can bind to Ca^2+^ binding enzymes and affect voltage-gated calcium channels, and therefore, lead to adverse biological effects (Sherry et al. 2009).

During MRI, tissues are pulsed with radio frequency in the presence of a magnetic field. This induces excitation of protons within water molecules. The release of energy when the protons relax back to their ground state is recorded, producing an MR image. Varying tissue signal intensity is determined by the relaxation time (T1 and T2) and proton density. GBCAs function to reduce the relaxation times increasing the signal intensity.

The primary categories of GBCAs are macrocyclic and linear. Gadobutrol, Gadoterate meglumine, and Gadoteridol are macrocyclic GBCAs, and the remaining are linear forms. Macrocyclic GBCAs form cage-like structures with Gd^3+^ enclosed in the cavity of the complex. The macrocyclic GBCAs tend to have lower dissociation constants and are therefore thought to be more stable than the linear GBCAs. The higher the dissociation constant, the more likely free gadolinium can be released into the circulation and tissues (Port et al. 2008). The GBCAs (both linear and macrocytic) can be ionic (dissociates into charged particles in a solution), nonionic, nonspecific extracellular, and tissue-specific.

Sieber et al., conducted in vivo studies in rats to compare the potential for the development of NSF between different GBCAs (Sieber et al. 2008a). Their data suggest that GBCAs differ in the potential to release Gd^3+^ into the skin. They observed fibrosis, increased cellularity, and increased cell swelling in 80 % of animals treated with Omniscan (a non-ionic, linear GBCA). The skin lesions appeared to correlate with high Gd^3+^ concentrations in the skin, liver, and femur.

In a review by Morcos (2008), it was determined that studies in rodents with normal renal function demonstrate that Gd^3+^ tissue retention was greater after injection with a non-ionic linear GBCA (gadodiamide) when compared to an ionic linear GBCA (gadopentetate). The lowest level of tissue retention occurred with a non-ionic macrocyclic agent (Gadoteridol). This similar observation (more deposition with a linear GBCA) was made by Gibby et al. who studied the retention of gadolinium in human bone tissue collected from patients undergoing hip joint replacement surgery (Gibby et al. 2004).

## Gadolinium tissue accumulation

Chelated Gd^3+^ has been thought to be swiftly cleared by the kidneys in those with normal renal function. However, studies in animals and humans have revealed that Gd^3+^ (free or chelated) is retained in a number of tissues in those without renal impairment. In rats repeatedly administered linear GBCAs, Gd^3+^ deposition in the cerebellum was observed, as well as progressive and persistent T1 signal hyperintensity (Robert et al. 2015). Healthy rats were administered IV injections of 0.6 mmol/kg of a GBCA or saline 4 times per week for 5 weeks (a total of 20 injections). T1-weighted MRI was performed before injection, once a week during the administration of compounds, and 4 weeks after the administration period. Inductively coupled, plasma mass spectrometry was used to measure total gadolinium concentration. The linear GBCAs studied induced increased signal intensity in the deep cerebellar nuclei. By Week 10, the total gadolinium concentration in the cerebellum of rats treated with linear GBCAs was higher than in rats treated with a macrocyclic GBCA.

Using inductively coupled plasma mass spectroscopy, Kanda et al. evaluated gadolinium accumulation in post-mortem brain tissues of subjects who received GBCAs but who were not diagnosed with any renal disease (Kanda et al. 2015a). They found that gadolinium accumulated in the brain, particularly in the dentate nucleus and globus pallidus. Administration of GBCAs is also associated with increasing signal intensity within the dentate nucleus and globus pallidus on unenhanced T1-weighted MRIs (Stojanov et al. 2016). In a number of studies by different researchers, signal intensity ratios between the dentate nucleus and globus pallidus were compared to control regions via retrospective analyses on T1-weighted MR images. GBCA deposition in the dentate nucleus and globus pallidus appears to depend on the GBCA class administered (Kanda et al. 2015b; Radbruch et al. 2015) and is also dose dependent (Kanda et al. 2014).

Studies by Radbruch and Kanda demonstrate that the administration of the linear GBCAs is associated with higher signal intensities. Kanda et al. performed a longitudinal analysis of data of a group of 381 consecutive patients who had undergone whole-brain, contrast-enhanced brain MRI. Comparisons were made between patients who had 6 or more contrast-enhanced brain MRIs and those who had unenhanced MRI. The dentate nucleus and globus pallidus signal intensity ratios in patients who had undergone contrast-enhanced examinations were greater than those of patients who had undergone unenhanced examinations. The ratios also correlated with the number of previous GBCA administrations.

McDonald et al. also demonstrated the association of the administration of GBCAs in patients with normal renal function with the deposition of gadolinium in neuronal tissue. Comparisons were made of post-mortem neuronal tissue between subjects that received unenhanced MRI and those who received at least 4 enhanced MRIs. Increased levels of elemental gadolinium were detected in all studied neuronal tissues of all patients exposed to multiple GBCA administration, and the dentate nucleus contained the highest concentrations of the regions studied. T1-weighted signal intensity and tissue gadolinium concentrations were also found to be dose-dependent.

Murata et al. used inductively coupled plasma mass spectroscopy to analyze brain, bone, and skin tissue for gadolinium in subjects undergoing autopsy. Comparisons were made between those who had received macrocyclic or linear GBCAs (Murata et al. 2016). This group also found gadolinium deposition in normal brain and bone tissue of patients with normal renal function.

A study by Wang et al. demonstrated the deposition of gadolinium in the skin, bone, and liver in rats exposed to gadodiamide (a non-ionic linear GBCA) or gadoteric acid (an ionic macrocyclic GBCA) (Wang et al. 2015). The skin Gd^3+^ concentration was significantly higher (180-fold) in rats that received gadodiamide than in those treated with gadoteric acid. Significantly higher concentrations were also found in the femur and liver. In addition, there were more histological alterations (e.g., spindle and stellate cells under epidermis layer and thicker epidermis layer) in the skin of rats treated with gadodiamide rats compared with gadoteric acid treated rats.

Gadolinium deposited in the bone can persist long term. Femoral head bone samples were collected from patients who underwent total hip replacement surgery up to 8 years after GBCA exposure (Darrah et al. 2009). Gadolinium bone concentrations were compared between bones from those patients exposed to GBCAs and those who were not. High concentrations of gadolinium were observed in the bone tissue from GBCA-exposed patients even when taking into account the bone-incorporation of gadolinium from natural sources. The observed bone deposition was seen in bone collected even 8 years after GBCA exposure. The gadolinium deposition levels did not correlate with the amount of time that elapsed since exposure.

These studies demonstrate that gadolinium can accumulate in tissues regardless of renal function. Whether the accumulated gadolinium is free Gd^3+^ or chelated is not completely clear. However, administration of GBCAs with higher dissociation constants (i.e., linear GBCAs) is associated with higher tissue accumulation. Therefore, the possibility that the accumulated gadolinium is free Gd^3+^ should be strongly considered, as well as the potential toxicological effects.

## Gadolinium-based contrast agent toxicity data

Data linking GBCA exposure with toxicological endpoints (apart from NSF in individuals with severe renal impairment) is limited. Although the toxicity of free lanthanide ions is well known (Haley et al. 1961; Spencer et al. 1998, 1997; Yoneda et al. 1995), the mechanism of GBCA toxicity in people with renal impairment is not fully elucidated. Further, the mechanism of gadolinium dissociation and subsequent exposure of tissues to free Gd^3+^ is not well described. Reports from patients describing adverse effects after repeated GBCA administration and study results (in vitro, in vivo animal, and human case reports, see Table 1) provide data that demonstrate the need to further investigate the potential toxicity of GBCA exposure in those with normal renal function.

**Table 1:** GBCA-induced toxicity endpoints (excluding NSF)

Toxicity endpoints	Study/report type	Species/cells	Reference
Necrosis and apoptosis	In vitro	Renal tubular cells	Heinrich et al. (2007)
Nephrotoxicity (reduced glomerular filtration rate)	In vivo	Pigs	Elmstahl et al. (2006)
Nephrotoxicity (acute tubular necrosis)	Case report	Human	Akgun et al. (2006)
Hematoxicity (reduced WBC count) Hepatotoxicity (vacuolar degeneration, disorganized hepatic cords)	In vivo	Mice	Chen et al. (2015)
Pancreatitis	Case report	Human	Blasco-Perrin et al. (2013)
Neurotoxicity (myoclonus, ataxia, tremor, and corpus callosum damage and hemorrhage)	In vivo	Rats	Ray et al. (1996)
Neurotoxicity (encephalopathy)	Case report	Human	Hui and Mullins (2009)

A number of studies have demonstrated adverse effects associated with GBCA exposure or administration. Induction of necrosis and apoptosis by GBCA exposure has been demonstrated in exposed renal tubular cells in vitro (Heinrich et al. 2007). In a study by Heinrick et al., confluent proximal tubular epithelial cells (LLC-PK1 cells) were incubated for 24 h with either control media (serum-free M199) or different contrast agents, including GBCAs, at concentrations used for angiography. GBCAs induced higher cytotoxicity than iodinated radiographic contrast media. Necrosis and apoptosis was induced by gadopentetate dimeglumine and apoptosis was observed in cells exposed to gadoterate meglumine.

Results from an in vivo study in pigs also demonstrate the higher toxicity of GBCAs when compared to iodine contrast agents (Elmstahl et al. 2006). Pigs were injected, via the right renal artery, with various concentrations of iodine contrast media (Mannitol/iohexol) or GBCAs (gadopentetate dimeglumine, gadodiamide). Gadopentetate and gadodiamide had higher nephrotoxicity (reduced glomerular filtration rate) than iohexol. The acute nephrotoxic effect of GBCA administration to humans has been demonstrated in a case report by Akgun et al. A 56-year-old woman with normal baseline renal function had 2 consecutive vascular imaging procedures employing GBCA administration. A few days later, the patient developed acute renal failure. A renal biopsy revealed acute tubular necrosis (Akgun et al. 2006).

The effects of GBCA administration are not limited to nephrotoxic effects. Histopathological and molecular changes (apoptosis) in the liver, lungs, and kidney tissues have been observed in GBCA-treated mice (Chen et al. 2015). In a study reported by Chen et al., Balb/c mice were dosed IV with different contrast agents, including gadopentetate dimeglumine. The studied GBCA induced a number of changes including reduction in total white blood cell count, increases in serum levels of inflammatory cytokines (IL-6 and TNF-R), and hepatic histopathologic changes (vacuolar degeneration, disorganized hepatic cords). Gadolinium-induced recurrent acute pancreatitis was reported in a 58-year-old woman administered gadobenate dimeglumine for MRI (Blasco-Perrin et al. 2013). Pancreatitis ensued approximately 3 h after initial GBCA exposure and upon repeated dosing. Another case report demonstrates the onset of pancreatitis after GBCA administration (Erenoglu et al. 2007). A previously asymptomatic 45-year old woman experienced upper abdominal pain and vomiting 4 h after cranial MRI with gadodiamide. Twelve hours after the onset of her abdominal symptoms, an MRI was performed that revealed severe necrotizing pancreatitis requiring surgical intervention.

The neurotoxic potential of GBCA administration has also been demonstrated. In a study to determine the potential for gadolinium-induced neurotoxicity, male rats received gadopentetate dimeglumine via injection into the lateral ventricle. This produced acute neurotoxicity manifested as myoclonus, ataxia, and tremor. Behavioral effects and morphological abnormalities (hemorrhage and damage to the corpus callosum) were also induced (Ray et al. 1996). Hui reports the development of encephalopathy in a woman who received a GBCA for MRI (Hui and Mullins 2009). Mass spectrometry detected 23,000 nmol/mL of gadolinium in a CSF sample obtained from the patient.

## Review of gadolinium biochemical and molecular mechanisms of action

Significant data regarding brain and other tissue accumulation of gadolinium are now available. However, the clinical significance of this phenomenon is not fully understood. This effort can be facilitated by a better understanding of the mechanisms associated with the development of gadolinium-induced toxicological endpoints such as NSF. Mechanistic data is crucial to determine therapeutic and preventive measures to treat and reduce the risk of adverse events associated with GBCA exposure.

Various studies (summarized in Table 2) have suggested the role of apoptosis, oxidative stress, transmetallation, and competition of Gd^3+^ with Ca^2+^ for cellular processes. In a review by Idée, dechelation leading to the release of free Gd^3+^ may lead to the release of chemokines and subsequent attraction of CD34 + fibrocytes followed by the development of fibrosis (Idee et al. 2014). Galdo et al. also attributes the release of chemokines to the pathogenesis of NSF (Del Galdo et al. 2010). Activated macrophages were exposed in vitro to 50 mM gadodiamide. This induced significant increases in chemokine gene expression. The chemokine gene expression was found to be dependent on NFkappaB activation.

**Table 2:** Potential mechanism of gadolinium toxicity

Mechanisms	Study type	Test subjects/cells	Reference
Release of chemokines and subsequent attraction of CD34 + fibrocytes leading to fibrosis	In vitro	Human macrophages	Idee et al. (2014) Del Galdo et al. (2010)
Stimulation of the expression and release of the cytokines involved in tissue fibrosis development	In vitro	Human monocytes	Newton and Jimenez (2009)
Induction of expression of a profibrotic chemokines and cytokines: IL-4, IL-6, IL-13, and VEGF in monocytes and type I and II collagen in fibroblasts	In vitro	Human monocytes Human fibroblasts	Wermuth and Jimenez (2014)
Inhibition of stretch-activated and voltage-gated calcium channels Blockage of Ca^2+^-dependent enzymes such S-transferases, dehydrogenases, kinases, ATPase, and glutathione Disruption of Ca^2+^ homeostasis	In vitro	Rat and human cells Isolated rat atrium Rat cortical neurons	Mlinar and Enyeart (1993) Laine et al. (1994) Xia et al. (2011)
Induction of fibronectin expression, apoptosis, and necrosis in fibroblasts Induction of fibrocyte markers (CD34 and procollagen type I)	In vitro In vivo	Human foreskin fibroblasts Rats	Do et al. (2014)
Mobilization of iron and the differentiation of peripheral blood mononuclear cells into ferroportin-expressing fibrocytic cells	In vivo	Mice	Bose et al. (2015)
Apoptosis	In vitro	Alveolar marcrophages Rat cortical neurons Hepatocytes	Mizgerd et al. (1996) Xia et al. (2011) Liu et al. (2003)
Elevation of reactive oxygen species	In vitro	Rat cortical neurons Mitochondria	Xia et al. (2011) Liu et al. (2003)
Blockage of ATP and ADP hydrolysis via stimulation of angiotensin II AT1 receptors	In vitro	Rat aortic rings	Angeli et al. (2011)
Effects on ACE activity via transmetallation with zinc	In vitro In vivo	Rabbit lung ACE Rats	Corot et al. (1998)

Newton and Jimenez studied the role of macrophages in NSF pathogenesis. There may be a direct profibrotic and proinflammatory effect of the chelated gadolinium in macrophages. They review the results of studies conducted in human peripheral blood monocytes exposed to varying concentrations of GBCAs or GdCl_3_. Real-time PCR was used to determine gene expression of various cytokines. Gadodiamide and gadopentetate were found to stimulate the expression and release of the cytokines involved in the development of tissue fibrosis.

Wermuth and Jimenez also studied the effects of GBCAs treatment on monocytes with the objective of measuring the levels of various interleukins, TGF-β, and vascular endothelial growth factor (VEGF) in the gadodiamide-treated monocytes (Wermuth and Jimenez 2014). They also treated human dermal fibroblasts with conditioned media from monocytes exposed to GBCAs. They observed increased IL-4, IL-6, IL-13, and VEGF expression in the GBCA-treated monocytes. Types I and II collagen expression was elevated in fibroblasts that were cultured with monocyte supernatant. Their data suggest that GBCAs (regardless of chelate class) induce expression of a number of profibrotic chemokines, cytokines, and growth factors in normal human monocytes. Therefore, GBCA stability may not be the main factor associated with the development of NSF.

One well-recognized factor related to the toxicity of Gd^3+^ is its size similarity and resulting competition with Ca^2+^ in cellular and biochemical processes. It is capable of inhibiting stretch-activated and voltage-gated calcium channels. As such, Gd^3+^ can cause inhibition of vital physiologic processes associated with (but not limited to) muscle tissue and neurons. It can also block Ca^2+^-dependent enzymes such S-transferases, dehydrogenases, kinases, ATPase, and glutathione (Laine et al. 1994; Mlinar and Enyeart 1993; Ramalho and Semelka 2015).

Do et al. provides data that suggest that the induction of fibronectin expression in fibroblasts is involved in the pathogenesis of NSF (Do et al. 2014). The in vitro (human foreskin fibroblasts) and in vivo (rats) consisted of exposure of the test subjects to GBCA with low (gadodiamide) or high (gadoteridol) thermodynamic stability constants. Gadodiamide induces fibronectin expression in the cultured fibroblasts. Also, gadodiamide appears to cause both apoptosis and necrosis in the fibroblasts. For the rat study, fibrocyte markers (CD34 and procollagen type I) are induced by GBCAs in skin. Both gadodiamide and gadoteridol had similar toxicology profiles and led to proximal tubule vacuolization. The data suggest that chelates with low thermodynamic stability constants are more likely to induce NSF.

Studies by Bose et al., indicate that iron participates in GBCA-toxicity mechanisms. Gadolinium-based contrast agent administration induces mobilization of iron and differentiation of peripheral blood mononuclear cells into ferroportin-expressing fibrocytic cells (Bose et al. 2015). In chronic renal disease–induced mice injected with gadodiamide, iron chelator therapy with deferiprone was found to prevent gadodiamide-induced NSF. This was evidence by a decrease in skin lesions such as redness, hair loss, and ulceration, and these effects were confirmed via histopathological analysis. Biopsy measurements showed reduced skin thickness in mice treated with gadodiamide and deferiprone when compared with the gadodiamide-only treated mice. There was also a reduction in markers CD163 and procollagen after deferiprone treatment. An increase in cells positive for ferroportin was observed with gadodiamide injections; this was reduced with deferiprone treatment.

Deferiprone treatment inhibited gadodiamide-induced differentiation of peripheral blood mononuclear cells into fibrocyte-like cells. It also decreased the release of catalytic iron by cells treated with gadodiamide. This data suggest that iron is involved in the pathogenesis of NSF in mice with chronic renal failure.

Mizgerd et al. studied the effects of on rat alveolar macrophages in vitro (Mizgerd et al. 1996). Different concentrations of gadolinium trichloride was added to the cell wells and incubated for different durations (0, 8, 16, or 24 h). Morphological, DNA, and flow cytometry data showed evidence of apoptotic cell death in the treated cells. Electron spectroscopy confirmed intracellular localization of gadolinium. Another role of apoptosis in gadolinium toxicity is its induction in hepatocytes (which also occurs subsequent to lanthanum and ytterbium exposure). This induction of apoptosis in hepatocytes may be related to elevated levels of reactive oxygen species (Liu et al. 2003).

Gadolinium chloride causes in vitro neurotoxicity. Xia et al. conducted in vitro studies with neurons from rat brains (Xia et al. 2011). The neurons were treated with gadolinium chloride for different time periods. The gadolinium treatment reduced neuron cell viability and disrupted intracellular calcium homeostasis. As in studies with a human hepatocyte cell line, gadolinium causes an elevation in reactive oxygen species levels. Expression levels of endoplasmic reticulum stress genes (e.g., XBP1) were analyzed and found to be elevated in gadolinium-exposed cells. Furthermore, N-acetylcysteine pretreatment inhibits gadolinium-induced cell death and endoplasmic reticulum stress further indicating a role of reactive oxygen species in gadolinium-induced toxicity.

Gadolinium blocks ATP and ADP hydrolysis, increases the vascular reactivity of rat aortic rings to phenylephrine via stimulation of angiotensin II AT1 receptors, and reduces nitric oxide bioavailability (Angeli et al. 2011). Incubation of rat aortic rings in 3 µM gadolinium results in an increase in ATP-induced vasorelaxation, indicating a reduction in ATP hydrolysis. The data from this study suggests that gadolinium induces endothelial dysfunction in rat aortic segments of rats by activation of ACE. This then leads to the release of vasoconstrictors from the endothelium. Corot et al. investigated transmetallation between marketed GBCAs and zinc-dependent angiotensin-converting enzyme (ACE). Both in vitro (rabbit lung ACE) and in vivo (rats) studies were conducted to determine the differences in the inhibition of ACE activity between the various GBCA forms. The in vitro studies showed a dose-dependent inhibition of ACE activity by the two linear GBCAs studied. For rats injected with a given GBCA, ACE activity was assayed in plasma, and the urinary zinc excretion was measured. The results for these studies showed inhibition of ACE by the linear, but not the macrocyclic GBCAs (Corot et al. 1998).

A 55-year-old man developed myelopathy, and progressive neurologic symptoms including hand and foot numbness, gait ataxia, and weakness in the extremities. A year after the onset of neurologic symptoms, serum zinc levels were found to be elevated and a diagnosis of idiopathic zinc toxicity was made. During the evaluation periods, the patient underwent MRI imaging examinations using gadopentetate dimeglumine. Urine analyses of several elements (including gadolinium) were performed periodically during chelation therapy to monitor zinc removal. After 2 years of periodic chelation therapy, gadolinium retention was evident. It was also found that the patient had long-term use of a dental product that contained high levels of zinc. Therefore, the chronic zinc toxicity contributed to tissue retention of gadolinium due to transmetallation (Greenberg 2010).

Data from studies by Taupitz suggest that competitive chelation with the glycosaminoglycan (GAG) heparin in the extracellular matrix may affect tissue distribution and toxicity of GBCAs (Taupitz et al. 2013). The researchers incubated three linear GBCAs (Omniscan, OptiMARK, and Magnevist) for 2 h in heparin at 37 °C in the presence of ZnCl_2_. This led to an increase in T1-relaxivity for each GBCA. Also, incubation of three macrocyclic complexes (Gadovist, Dotarem, and Prohance) in heparin led to minor increases in T1-relaxivity. However, no differences in relaxivity between linear and macrocyclic GBCAs were observed when ZnCl2 was not present. It is possible that transchelation of Gd^3+^ from GBCA complexes to GAGs may contribute to Gd^3+^ retention and NSF-related inflammatory processes.

## Toxicity of other lanthanide complexes

In light of the modest amount and still emerging data regarding the toxicity of gadolinium complexes, it is useful to examine toxicity data regarding other lanthanide series elements given their chemical and physical similarity to gadolinium. A number of lanthanide series element complexes have been studied and found to deposit in tissues and contribute to toxicity. Nanoceria infusion in rats is associated with an increase in hepatic apoptosis (Tseng et al. 2014). Tseng et al. performed studies to determine the toxicologic potential of nanoceria given the human industrial exposure and its development as a therapeutic agent. Rats were infused with nanoceria (or water) via femoral vein cannula. The rats were sacrificed at different time points up to 90 days after the infusion. Histopathological, immunohistochemistry, serum biochemistry, and electron microscopy analyses were performed on rat tissues. The ceria caused Kupffer cell surface bulging and cellular enlargement. Granulomata were seen in rats sacrificed 30 and 90 days post infusion. Hepatic cell apoptosis and liver cell proliferation increased subsequent to nanoceria infusion. Gadolinium also affects Kupffer cells and may have other hepatic effects.

Europium-doped gadolinium oxide nanotubes (Gd2O3:Eu^3+^) has the potential for use as a multimodal contrast agent because of its superparamagnetism and T1 relaxation characteristics. Since these can accumulate in the bone, Jin et al. studied these nanotubes for their potential to adversely affect bone marrow stromal cells (BMSCs) (Jin et al. 2015). Europium-doped gadolinium oxide nanotubes were added to female mouse BMSC cultures for 24 h. Cell viability, apoptosis, and membrane integrity were assessed. Mitochondrial membrane potential, lysosome permeability, and reactive oxygen species measurements were also made.

Cell viability was adversely affected by Gd2O3:Eu^3+^, and the percent of apoptotic and late necrotic cells increased with Gd2O3:Eu^3+^ treatment. There was also loss of BMSC membrane integrity associated with Gd2O3:Eu^3+^ treatment. Mitochondrial damage was involved in Gd2O3:Eu3 + nanotube-induced necrosis in BMSCs, and exposed cells underwent substantial DNA damage. GBCAs are also found to accumulate in bone tissue. Therefore, the potential effects on BMSC should be considered and investigated.

Samarium, another lanthanide series element, is also toxic in its free ionic form. Samarium nitrate elicits reproductive toxicity in male mice treated for 90 days (Zhang et al. 2014). Specifically, the treatment was associated with disorganized seminiferous tubules, reduction in spermatogenic cells, and decreased sperm count. It appears that the testis may be a target organ of samarium toxicity, while gadolinium has various effects including nephrotoxicity and neurotoxicity.

## Discussion

There is significant in vitro, animal, and human data available that indicates the potential for adverse effects in people who have multiple or recurrent exposures to GBCAs. Studies over the years have demonstrated the possible mechanisms that contribute to some GBCA-induced endpoints such as NSF. Although gadolinium deposition is now well established to occur despite normal renal function, it is not known if the gadolinium retained in tissue is in the chelated or free ion form. Prospective studies incorporating measurement of serum and urine Gd^3+^ levels can help correlate the Gd^3+^ body burden with MRI T-weighted intensity data. Detection of persistent Gd^3+^ that is higher than background for those exposed to GBCAs will provide more insight into the association of abnormal health effects that occur subsequent to GBCA exposure. Retrospective cohort studies will also add a valuable set of data and information to determine adverse effects potential of GBCAs. This information along with the growing body of mechanistic data can allow the development of therapeutic and preventative measures.

The results of a patient advocacy group-initiated survey of 17 patients (Gadoliniumtoxicity.com) show the onset of a group of symptoms within a month of their last MRI (Gadolinium Toxicity: A Survey of the Chronic Effects of Retained Gadolinium from Contrast MRIs 2015). These symptoms range from neurological, musculoskeletal, to dermal, including pain (reported by 100 % of patients surveyed), muscle symptoms reported by 88 %, and ocular symptoms (reported by 76 %). For each additional symptom category (e.g., dermal, cognitive, ENT), more than 50 % of those surveyed reported symptoms in each of these and other categories. Notably, 15 of the 17 patients surveyed had urinary gadolinium levels above expected levels for patients with normal renal function (reference range is 0.0–0.4 mcg Gd/24-h). This information can serve as a guide for retrospective and prospective studies to determine associations between multiple GBCA administration and adverse health effects.

Studies regarding specific cytotoxic and other adverse outcomes from non-gadolinium lanthanide elements can also be considered when assessing the toxicity potential of GBCAs. Cerium, samarium, and europium have very similar biologic and chemical characteristics to gadolinium. These affect some of the same biochemical/molecular processes such as induction of apoptosis and necrosis, oxidative stress, and competition with Ca^2+^. Comparisons between the level of tissue retention (such as that measured in biopsied tissues when feasible) and urine gadolinium level, particularly in those who receive repeated GBCA administration, can also provide additional data and insight regarding tissue sources of free Gd^3+^. This may be done by measuring the level of gadolinium excretion over time and concurrently measuring the level of tissue gadolinium–deposit detection via biopsy if feasible (Thakral et al. 2007; Xia et al. 2010) or the use of T1-weighted MRI to determine if the gadolinium tissue-level changes correspond to excretion levels.

Studies to distinguish between chelated and Gd^3+^ levels and considering the mechanistic data is necessary to determine the best therapeutic and preventive measures for GBCA-related health effects. Thus far, it has been determined that deferiprone protects against GBCA-induced NSF in mice. If GBCA administration is the best option for a given patient, a pretreatment approach that protects against toxicological endpoints may be a viable option. A compound that can effectively compete with Gd^3+^ may be an approach to reduce any Gd^3+^ retained by the body. N-acetylcysteine pretreatment inhibits gadolinium-induced cell death and endoplasmic reticulum stress in vitro. It also protects against GBCA-induced nephrotoxicity in rats with chronic renal failure (Pereira et al. 2012).

Chelators used for iron chelation therapy may have efficacy for gadolinium chelation. Examples are deferoxamine, deferiprone, and deferasirox. In a case report of a 65-year-old woman with confirmed NSF, deferoxamine was administered intramuscularly at 500 mg/day, and then 1 week later at 1000 mg/day. While deferoxamine doubled urinary excretion of gadolinium, serum levels did not reduce significantly. The authors estimated that at the deferoxamine-induced increased rate of excretion, it would still take 78–156 years to rid the patient of all gadolinium. Therefore, a search for a stronger chelator was recommended (Leung et al. 2009). Chelators for mercury and lead toxicity can be investigated to determine the most efficient option for the chelation of rare earth elements (Flora and Pachauri 2010). Nanoparticles linked to chelators have been proposed as a treatment for Alzheimer disease to remove metals from neural tissue. This could serve as another approach to reduce the level of Gd^3+^ in various tissues, especially given the ability to cross the blood–brain barrier to chelate metals in brain tissue, followed by clearing the tissue of the nanoparticle-chelator-metal complexed molecule (Liu et al. 2005).

## Conclusions

Unequivocal data regarding the effects of multiple GBCA exposure are limited. However, the information regarding the thermodynamic stability constants for GBCAs, in vitro, animal, and human data, and the emerging data regarding gadolinium tissue accumulation in those with normal kidney function indicate that the potential toxicity associated with GBCA must be seriously and urgently considered. This concept must be addressed with retrospective and prospective cohort studies. Research providing additional mechanistic data is also paramount and will provide valuable information regarding how to prevent GBCA-related toxicity, treat existing GBCA-related health issues, guide the use of existing GBCAs, and direct the design of safer MRI contrast agents.
